# Reducing screen-time and unhealthy snacking in 9–11 year old children: the Kids FIRST pilot randomised controlled trial

**DOI:** 10.1186/s12889-020-8232-9

**Published:** 2020-01-29

**Authors:** Natalie Pearson, Stuart J. H. Biddle, Paula Griffiths, Lauren B. Sherar, Sonia McGeorge, Emma Haycraft

**Affiliations:** 1 School of Sport, Exercise & Health Sciences, National Centre for Sport & Exercise Medicine, Loughborough University, Loughborough, Leicestershire, LE11 3TU UK; 2 Institute for Resilient Regions, University of Southern Queensland, Springfield, Australia

**Keywords:** Screen-time, Energy-dense snacks, Families, Children, Intervention, Feasibility

## Abstract

**Background:**

Many young people form unhealthy behavioural habits, such as low intake of fruit and vegetables, high intake of energy-dense snack foods, and excessive sedentary screen-based behaviours. However, there is a shortage of parent-and home-focused interventions to change multiple health behaviours in children.

**Methods:**

Kids FIRST was a 12-week, home- and school-based pilot randomised controlled trial to reduce screen-time and unhealthy snacking with assessments at pre- (baseline) and post-intervention. Four UK schools were randomised to control or one of three interventions targeting reductions in (1) screen-time and unhealthy snacking (ST + Sn), (2) screen-time (ST only), (3) unhealthy snacking (Sn only), and parents with children aged 9–11 years were recruited via schools. Intervention group parents received four online ‘sessions’ and four packages of resources tailored to each group. Children received four 30-min lessons during school time. Children and parents reported their own screen-time behaviours, children reported their own snacking behaviours. Descriptive analyses were undertaken using principles of intention to treat.

**Results:**

Initial feasibility was shown in that this study successfully recruited schools and families into all four study arms and retained them over a period of 13 weeks (retention rate ≥ 74%). Seventy-five children and 64 parents provided full baseline questionnaire data. Reductions in children’s school day and weekend day TV/DVD viewing and computer game use were found in the ST + Sn and ST groups, while self-reported smartphone use increased in these groups. Similar results were found for parents’ TV/DVD, computer and smartphone use in these groups. Little to no changes were found in reports of the dietary variables assessed in any intervention group for children or parents.

**Conclusions:**

These preliminary findings show some promise for the Kids FIRST intervention. Based on these findings, a future full trial should recruit a more diverse sample of families and optimise the intervention and intervention resources to more fully engage parents with the dietary-based components of the intervention programme, where fewer changes were seen. Although most parents reporting receiving the intervention resources, further development work is required to achieve higher levels of engagement. This might include greater parent and child engagement work early in the development of the project.

**Trial registration:**

Retrospectively registered in June 21st 2019 with ClinicalTrials.gov (number NCT03993652).

## Introduction

During childhood, many young people form unhealthy behavioural habits, such as low intake of fruit and vegetables, high intake of energy-dense snack foods (e.g. chocolate, biscuits), and excessive sedentary screen-based behaviours (e.g. TV viewing and computer use). Such behaviours have been associated with poor health outcomes among children such as overweight and obesity, cardio-metabolic risk, and poorer mental health [1]. These behaviours have been shown to persist into adulthood [2–4], meaning that once unhealthy behaviours are established it is likely they will remain given the difficulty in changing behaviours later in life. This tracking of behaviours exacerbates the poor health outcomes associated with unhealthy behaviour and highlights the need for interventions which prevent excessive unhealthy lifestyle behaviours in childhood.

A key property of dietary behaviours and sedentary screen-time is that they often co-occur as risk behaviour clusters [5–7]. A variety of mechanisms may account for the synergy between screen-time and eating behaviours, including one behaviour serving as a stimulus to the other. For example, during time spent sitting in front of the TV and computers, children are exposed to numerous advertisements (most often for ‘junk foods’) that can influence the type of food desired, requested and consumed [8]. Furthermore, screen viewing behaviours may cause distraction resulting in a lack of awareness of actual food consumption or overlooking food cues, which may lead to overconsumption and increased energy intake [9]. Given this behavioural interconnectedness, it is plausible that reductions in unhealthy snacking behaviours may be equally well addressed by considering co-existing behaviours such as screen-time and vice versa. There is therefore a need to test the feasibility of interventions which promote reductions in both screen-time and unhealthy dietary behaviours reciprocally, whilst simultaneously comparing interventions which target the individual health behaviours.

While historically interventions have targeted individual health behaviours [10, 11], there has been a recent trend towards interventions which aim to modify several behaviours in young people simultaneously, including sedentary screen time and aspects of diet [12]. For example, interventions targeting screen-time and dietary behaviours in 7–10 [13] and 8–10 [14] year olds have shown some promising results for reducing screen-time [15, 16] and servings of unhealthy snacks [16] and for promoting fruit and vegetable consumption [15]. No evidence to date has examined an intervention which aims to compare the targeting of multiple and individual behaviours within the same study. Systematic reviews and meta-analyses show equivocal evidence surrounding the effectiveness of single versus multiple health behaviour interventions [17–20]. The contrasting findings between recent reviews may be due to the fact that some studies target both behaviours together (i.e. screen-time and diet) and other studies only target one behaviour (e.g. screen-time only). It is possible that the individual behaviours are targeted differently from how they are targeted in multiple behaviour interventions. While offering interventions with the contextually compatible targets of reducing screen-time and unhealthy dietary behaviours is practical in a public health context, further evidence is required to compare individual versus multiple health behaviour interventions within the same study.

While there is debate on single versus multiple health behaviour interventions for reducing screen-time and unhealthy dietary behaviours, there is much consensus that having parental involvement and targeting the home environment are key aspects relating to intervention success [15, 16]. Parents and caregivers, as well as the home environment, play a central role in the socialisation and development of health behaviours. This is evidenced by research linking parents’ own screen-time and dietary behaviours, parenting practices, and home availability and accessibility of screens and types of foods to children’s screen-time and dietary behaviours [21, 22]. A recent review concluded that there is a need for interventions which involve a parent more than just in a supervisory or administrative role [15] and that parents need to be active participants in interventions so as to maximise the chances of promoting successful behaviour change in their children [23]. Given that both screens and unhealthy eating behaviours are highly pervasive in today’s society, placing the task of reducing these behaviours solely on the child may be less effective than targeting the parent and child together. There is, however, a shortage of research focusing on how to target and involve parents to help their children engage in less screen-time and reduce their unhealthy dietary behaviours.

Children receive a great deal of their health behaviour messages via schools. Moreover, dietary [24] and sedentary behaviour [10] interventions have been successfully implemented in schools and schools are an effective way to deliver consistent intervention messages (i.e. this method typically has high fidelity). There is therefore value in involving schools as a component of successful family-based interventions, so as to provide an additional forum through which behaviour change messages can be delivered and reinforced.

Given the evidence reported above, the aims of this paper were to report on the feasibility and potential efficacy of the Kids FIRST (**F**amily-based **I**ntervention to **R**educe unhealthy **S**nacking and screen-**T**ime) four-arm family- and school-based intervention to reduce screen-time and/or unhealthy snacking in children aged 9–11 years.

## Methods

The Kids FIRST intervention was performed and reported in accordance with the Consolidated Standards of Reporting Trials extension to randomised pilot and feasibility trials guidelines [25], and the TIDieR (Template for Intervention Description and Replication) Checklist. The study was approved by the Ethical Advisory Committee of Loughborough University (R15-PO36).

### Setting and recruitment

Kids FIRST was family based with a school component. The study targeted families with at least one 9–11 year old child. Families were recruited through schools in the East Midlands region of the United Kingdom between September and December 2015. Twenty-five primary schools were selected from a database of schools and school contacts, and headteachers were sent study information via email, which was followed-up with a telephone call, inviting them to be part of the project. Seven schools (28%) agreed to participate. Subsequently, all parents and/or caregivers of children aged 9–11 years (*n* = 407; year 5 and 6 of Primary School) were sent information sheets via the school outlining the study details and inviting them and their child to participate. Based on our extensive formative work (unpublished), the recruitment materials promoted a ‘free 12-week programme for parents to encourage healthy lifestyle behaviours at home’. The method of recruitment (i.e. recruiting schools from across the East Midlands in the first instance and then families within schools), was aimed at targeting families from a range of socio-economic backgrounds. Three schools were excluded from participating in the project due to insufficient numbers of families providing consent (*n* < 8 per school). In participating schools (*n* = 4), written informed consent was obtained from 75 parents (*n* = 75/320 parents of children aged 9–11 years) for themselves and on behalf of their child, and children’s verbal assent was obtained at the time of baseline data collection. The flow of families into and through the Kids FIRST intervention is displayed in the Consort Flow diagram (Additional file 1).

### Study design

The study was a pilot four-arm cluster randomised controlled trial with assessments at pre- (baseline) and post-intervention (13 weeks after baseline). The study arms were three intervention groups and a control group. The intervention groups were: Group 1: targeting reductions in screen-time and unhealthy snacking (ST + Sn), Group 2: targeting reductions in screen-time (ST), and Group 3: targeting reductions in unhealthy snacking (Sn). Following consent by headteachers, schools were randomised to either one of the three intervention groups or the control group by NP using computer generated random sequences. Only NP had access to the randomisation sequences (which were on a password protected file).

### Theoretical underpinning of the kids FIRST intervention

The Kids FIRST intervention was framed in a social ecological perspective [26], and was theoretically informed, drawing on constructs designed to address potential individual, behavioural, social and physical home environmental mediators derived from Habit Theory [27], Behavioural Choice [28], and Social Cognitive theories of individual behaviour change [29]. The content of the intervention drew heavily on our formative research with children, teachers and parents (unpublished) which examined, through focus groups and interviews, key factors that were deemed important in relation to screen-time and snacking behaviours, how these factors could be addressed and possible ways of structuring and delivering a family-based intervention targeting these behaviours.

The taxonomy of behaviour change techniques [30, 31] was applied to characterise the association between the potential mediators targeted in the Kids FIRST study, the intervention components / strategies developed, and the theoretical underpinning. Additional file 2: Table S1 describes the specific behaviours that were targeted by the intervention and the practical application of the behaviour change techniques applied in relation to the Kids FIRST intervention.

### Kids FIRST intervention overview

The intervention was implemented over a 12-week period from October 2015 to May 2016 (rolling recruitment of schools) following the baseline assessments. Families in each of the three intervention arms received the same structure of intervention, but the content was tailored to the targeted behaviour(s). Parents and children attended an introductory group session at school which provided an overview of the programme. Families in the control group did not receive any resources or engage with any sessions. No changes to the trial methods were implemented after the commencement of the programme.

#### Family setting

The intervention consisted of four blocks of 3 weeks. Each block targeted and focused on specific evidence-based mediators (Additional file 2: Table S2). All intervention families were given access to the Kids FIRST website (which was live for the duration of the study only), which included a login page where families were directed to the content/pages that were specific to their intervention group (i.e. families in group 1 could only access the webpages for group 1) by a group sensitive password. During each block, parents in each intervention group received one online session (delivered via PowerPoint or an audio file), and a package of resources (e.g. newsletters, information sheets, charts etc.) delivered via their child from school (see Additional file 2: Table S2). The newsletters and resources (e.g. monitoring charts, top tips, recipes, and alternative activity or snack ideas) aimed to support the key learning messages delivered in the online sessions and to the children in the classroom lessons (see below).

The key messages of the programme, which were reinforced at each block of the intervention, were:

- Increase knowledge about ST/Sn outcomes (health and other);
- Increase awareness and implementation of strategies to participate in healthy ST and/or consumption of healthy snacks, and
- Guide parents on how to implement behaviour modification, such as planning and monitoring as a means of empowering families to make behavioural changes that were specific to them.

#### School setting

Children randomised to an intervention received four 30-min lessons during school time over the intervention period (one per ‘block’). Class lessons were delivered by trained research personnel. Key learning messages incorporating key principles of behaviour change were delivered to whole year group classes. The class lessons were followed by homework activities/challenges and were aimed at the children and were designed to (i) target habits and self-efficacy, (ii) target screen-time and/or nutritional knowledge; (iii) introduce alternative activities/snacks, and (iv) encourage children to be positive role models to family and friends. In the process of developing resources, we engaged with *n* = 17 teachers to design class lessons prior to the commencement of the programme and incorporated literacy and numeracy aspects that were aligned to the national curriculum specific for each age group.

### Measurement and data management

Data were collected before (week 0) and after the intervention (week 13). At both time points, children received questionnaires in a sealed envelope to take home for completion by themselves and one of their parents/caregivers. Parents and children in each of the four arms received the exact same measurement protocol and completed the same measurements at both time points. No changes were made to the measurements were made after the commencement of the intervention.

### Child questionnaire

Children completed the questionnaire at home, and parents were asked to help their child if/when required. Children self-reported their age, date of birth and sex, and completed a range of self-report measures.

### Primary outcomes

#### Screen-time

Children reported the time (in hours and minutes) that they spent using three different types of screens on a usual school day and on a usual weekend day using an adaptation of the Adolescent Sedentary Activity Questionnaire (ASAQ) [32], which has been used successfully in this age group previously [33]. The adaptation allowed for days of the week to be categorised as school day and weekend days. Time spent at each type of screen was converted into minutes per school day and weekend day, respectively.

#### Eating behaviours

Frequency of food intake was assessed using the frequency scales of the Child Nutrition Questionnaire [34], which has been shown to be valid and reliable in children of this age. Children indicated how frequently they consumed portions of fruit, vegetables, savoury snacks, and sweet snacks on a usual day. Several examples of food types and portion sizes were given to children. Five response categories were available: (i) never/I don’t eat (fruit), (ii) less than one portion a day, (iii) 1–2 portions a day, (iv) 3–5 portions a day, (v) 5 or more portions a day. The frequency of food consumption was converted to a daily equivalent, which is an established method that has been used successfully in other dietary studies [35]. The frequency of consumption of the four food categories was converted to a continuous daily equivalents as follows: never (0 per d); less than one portion a day (0·5 per d); 1–2 portions a day (1·5 per d); 3–5 portions a day (4.0 per day); and 5 or more portions a day (5.0 per day).

### Secondary outcomes

#### Individual

Children were asked four questions about their habits for eating snack foods in front of the television using the valid Self-Report Behavioural Automaticity Index (SRBAI) [36], which has been successfully used in children of this age [35]. They were asked the same four questions regarding eating sweet and savoury snacks, eating fruit and vegetables as a snack, eating fruit and vegetables in front of the TV, and regarding habit for watching TV. Responses were made on a five-point Likert scale, ranging from [1] ‘strongly disagree’ to [5] ‘strongly agree’, and summed separately to provide five habit scores.

Using a scale previously designed for children of this age [37], children were asked six questions about their self-efficacy for reducing their energy-dense snack food consumption (i.e. snacks including chocolate, crisps, biscuits, and sweets (candy)). They were asked the same six questions about not eating snack foods in front of the TV, about eating more fruit and vegetables, and about reducing their screen-time. Responses were made on a five-point Likert scale, ranging from [1] ‘not at all sure’ to [5] ‘very sure’, and summed separately to provide four self-efficacy scores.

#### Behavioural

Children were asked how often they ate dinner while also watching TV during a typical week, using a previously designed questionnaire, that has been used in children of this age [38]. Response options were on a four-point Likert scale: (1) ‘never’, (2) ‘1-2 times a week’, (3) 3–6 times a week’, and (4) ‘every day’. Weekly equivalents for eating dinner at the TV were calculated as follows: never (0); 1–2 times a week (1.5); 3–6 times a week (4.5); and every day [7].

Using the above questionnaire [38], children were asked how often they ate sweet and savoury snacks, and fruit and vegetables while watching TV during a usual day. Responses were provided using a four-point Likert scale: (1) ‘never’, (2) ‘once a day’, (3) ‘1-2 times a day’ to (4) ‘3 or more times a day’. Daily equivalents for snacks and FV eaten at the TV were calculated as follows: never (0 per d); once a day (1.0 per d); 1–2 times a day (1·5 per d); and 3 or more times a day (3.0 per day).

#### Social environmental

Using previously designed scales [39], children were asked about the rules that their parents set at home regarding their screen-time and eating behaviours. Children were asked if parents set rules for (i) how long, (ii) when, and (iii) what they can watch (or use the computer for) on the television and computer, respectively. Children were asked if parents set rules for (i) how many/much, (ii) when they can eat, and (iii) the types of sweet/savoury snacks and fruit and vegetables they could eat, respectively. Children were asked if parents set rules for (i) how often, (ii) when they can eat, and (iii) what they can eat while watching the television or using a computer, respectively. Response options were ‘No’, ‘Sometimes’, and ‘Yes’. For the purpose of these analyses the response options ‘sometimes’ and ‘yes’ were combined into ‘yes’ and composite scores for each behaviour were created by summing responses (i.e. rules for how long, when, and what child can watch on TV were summed and divided by three to create ‘rules for TV use’).

#### Home environmental

Children were asked to report the number of televisions and computers they had access to at home, whether they had a television in their bedroom, and whether they had a computer (laptop, games console) in their bedroom.

Children were asked questions regarding the in-home accessibility of energy-dense snacks (two items) and of fruit and vegetables (four items) over the past week (e.g. ‘in the past week, were there any fruits that were prepared and ready for you to eat as part of a meal or snack?’). Responses were given on a three-point Likert scale: (1) No, (2) Sometimes, and (3) Yes. Scores for the two energy-dense snacks questions were summed to create the ‘home accessibility of energy-dense snacks’ score, and scores for the four fruit and vegetable questions were summed to create the ‘home accessibility of fruit and vegetables’ score.

### Parent questionnaire

Parents reported their relationship to the child that was enrolled in the Kids FIRST project, their own age, gender, ethnicity, marital status, level of education, home postcode, and their social status (using a subjective socioeconomic status scale [40]).

#### Parental screen time

Parents indicated the time (in hours and minutes) that they spent sitting at various screens (watching TV/DVDs, using a computer/tablet for work, using a computer/tablet for fun, and using a smartphone for the internet) on a usual week day and a usual weekend day using an adaptation of the domain-specific sitting questionnaire [41]. Time spent at each type of screen was converted into minutes per weekday day and weekend day, respectively.

#### Parental eating behaviours

Parental frequency of consuming fruits, vegetables, savoury and sweet snacks was assessed using the same frequency scales that children completed (see above). Consumption frequency of the four food categories were converted as outlined above.

### Process evaluation

After completing the intervention, all parents in the intervention arms were invited to complete a short follow-up survey that included a series of closed- and open-ended questions. Topics addressed were study sign-up; the introductory session; the Kids FIRST intervention (including: resources, website and home materials); perceptions of behaviour change; and Kids FIRST school-based lessons.

### Potential efficacy

Potential efficacy was assessed by taking measures of primary and secondary outcomes (described above) at baseline and post-intervention and comparing changes in the outcomes.

### Analyses

All statistical analyses were performed using STATA (Statacorp, TX). Analyses were conducted under the intention to treat (ITT) assumption by carrying baseline data forward, thus all efficacy analyses were conducted on a sample of 75 children (*n* = 21 in ST + Sn; *n* = 25 in ST only; *n* = 14 in Sn only, and *n* = 15 control) and 64 parents (*n* = 19 in ST + Sn; *n* = 22 in ST only; *n* = 12 in Sn only, and *n* = 12 control). Differences in means (and 95% CI) between baseline and follow-up were calculated as post-intervention mean minus baseline mean. As the data are from a feasibility trial, we are not powered to detect differences between groups, hence *p*-values are not reported. There were not sufficient schools within each cluster to be able to adjust for school clustering in the analysis (as is common practice with small studies, e.g. [42]) and so results should therefore be interpreted with caution.

Process evaluation responses to open-ended questions were transcribed verbatim. Statements for each question were then entered into Mindgenius V6.0 mindmapping software. The statements were inductively content analysed and grouped into coherent themes and sub-themes. Full statements were split into multiple statements, where appropriate. Numbers and percentages are reported for indicative purposes only and do not necessarily reflect importance. Closed-response questions requested yes/no or Likert-scaled responses, and appropriate descriptive statistics (percentages, frequencies, means, standard deviations) were used to describe such data.

## Results

### Recruitment and adherence

Seven schools (28%) agreed to participate, and four schools were eligible to participate (see CONSORT Flow Diagram, Additional file 1). Written informed consent was obtained from 75 parents (*n* = 75/320 parents of children aged 9–11 years) for themselves and on behalf of their child, and children’s verbal assent was obtained at the time of baseline data collection. Seventy-five children and 64 parents provided full questionnaire data at baseline and were randomised to one of the four study arms (see Additional file 1). Post-intervention measures were not completed by 12 children (16%) and 18 parents (26%). No differences in key variables were found between those who provided valid data at both time points compared with those who provided data at baseline only (data not shown). Table 1 presents the baseline characteristics of the child and parent samples.

**Table 1:** Descriptive characteristics of the child and parent participants at baseline

	Study arm			
	ST and Snacking (*n* = 21)	ST only (*n* = 25)	Snacking only (*n* = 14)	Control (*n* = 15)
Child sex (N (%))				
Male	10 (47.6)	12 (48)	7 (50)	6 (40)
Female	11 (52.4)	13 (52)	7 (50)	9 (60)
Child age (mean, SD)	9.90 (0.53)	9.84 (0.69)	9.86 (0.36)	9.73 (0.46)
	Study arm			
	ST and Snacking (*n* = 19)	ST only (*n* = 22)	Snacking only (*n* = 12)	Control (*n* = 12)
Parent sex (N (%))				
Male	3 (15.7)	4 (18.1)	1 (8.3)	2 (16.6)
Female	16 (84.3)	18 (81.9)	11 (91.7)	10 (83.4)
Parent age (mean, SD)	42.8 (6.41)	41.5 (8.53)	44.3 (2.56)	38.42 (5.43)
Ethnicity (N (%))				
White / White British	18 (94.7)	21 (95.4)	12 (100)	9 (75.0)
Other	1 (5.3)	1 (4.6)	0	3 (25.0)
Parental marital status (N (%))				
Married	14 (73.7)	18 (81.8)	9 (75.0)	7 (58.3)
Other	5 (26.3)	4 (18.2)	3 (25.0)	5 (41.7)
Parental education (N (%))				
A-Level or post A-level equivalent	9 (47.3)	8 (36.3)	5 (41.7)	7 (58.3)
Degree level or above	10 (52.7)	14 (63.7)	7 (58.3)	5 (41.7)
Social status (mean (SD); median = 7)	6.21 (1.44)	6.85 (1.18)	6.92 (1.08)	5.83 (2.04)

### Process evaluation

#### Recruitment and study sign-up

Parents expressed various reasons for signing up to take part in Kids Frist, including general interest (39%), motivation of the child to be involved (11%), and an interest in screen-time (11%) and healthy eating (7%). The introductory session at the school, for both parents and children, was seen as beneficial by 77% of the parents attending. Parents frequently stated that the most useful elements of the session were receiving advice, practical help, facts and messages.

#### Planned programme

The programme duration (13 weeks) was seen as acceptable by most parents (92%). Several resources were provided to parents throughout the duration of the intervention. The preferred method to receive these resources was via their child from school (85%), with 12% preferring it via email. Moreover, most parents and their families engaged with paper-based materials (93%) rather than via the website (1%) or email (6%). Parents reported whether they had received the resource, whether they had used it, and how useful they thought the resource was. Table 2 shows that the most used resources were the newsletters. Of those that received and used the resources, most parents reported them to be useful (Table 2). The most useful were the top tips sheets and newsletters. Although most parents reporting receiving the resources, it seems that further development work is required to achieve higher levels of engagement. This might include greater parent and child engagement work early in the development of the project.

**Table 2:** Description of resources received, used and found useful by intervention parents

	Received	Received but not used	Received and used	Those who received and used found the resource useful
Habit booklet	77	44	42	86
Recipe cards	60	54	32	59
AZ	81	42	42	71
Activity/Snack jar	57	54	27	57
Newsletters	81	32	63	98
Monitoring charts	79	48	30	82
Child activities	79	49	38	58
Top Tips	77	41	44	100
Goal setting activity	66	58	26	95

#### Kids FIRST website

A Kids FIRST website was created and four online information sessions were available for participants to access. Half reported accessing information on the website and 21% downloaded materials. For those accessing the site, most only did so for 1–2 sessions, but they did report that the information was useful (86% agreement). Most agreed that the number of online sessions was acceptable (85%).

#### Changing behaviours

There was reasonable agreement (63–73%) that after the study the parents felt they knew more about healthy and unhealthy habits, parents as role models, and the impact of home availability and accessibility. Fewer agreed that they knew more about home rules for behaviour change (58%) after the intervention (i.e. more ideas for rules that could be implemented). However, those who reported not knowing more about rules for behaviour change after the study felt that they already had that knowledge or were already implementing similar ideas and strategies in their family environments.

Just over half of parents (57%) felt that they had learned something new about unhealthy snacking and 67% reported that they had learned more about screen time. Given the results of this pilot study, and that 83% of parents felt that a decrease in their child’s screen time could be achieved, it appears that parents see screen time as more feasible for behaviour change than unhealthy snacking. This may be due to greater possibilities and perceptions of parental control.

#### School lessons

The project team delivered four lessons for children in school concerning reducing screen time and unhealthy snacking. Of those responding, 57% said that their child had talked to them about the lesson. Of these, 64% reported enjoying the lesson.

### Pre-post changes in outcome behaviours

Table 3 shows the mean time children spent in each of the screen-based activities at baseline and post-intervention. Reductions were seen for TV/DVD viewing and computer game use in some groups, and an increase in smartphone use was seen in some groups. However, the study was not powered or designed to examine whether these were significant.

**Table 3:** Children’s screen-time, and change in screen-time by study arm

Screen-time variables	Study arm	Mean (SD) minutes/day at baseline (week 0)	Mean (SD) minutes/day at post-intervention (week 13)	Difference in means (95% CI)
School day TV/DVD viewing	ST and Snacking	198.00 (169.82)	168.33 (255.43)	−27.25 (−92.85, 38.35)
	ST only	166.20 (193.34)	130.76 (138.05)	−35.44 (− 107.35, 36.47)
	Snacking only	118.57 (155.02)	139.61 (168.50)	19.61 (−112.43, 151.66)
	Control	119.13 (142.26)	123.80 (99.00)	4.66 (−85.52, 94.85)
Weekend TV/DVD viewing	ST and Snacking	215.90 (141.89)	160.61 (105.46)	−47.25 (−88.33, −6.16)
	ST only	161.48 (107.74)	147.88 (103.44)	−13.60 (−74.61, 47.41)
	Snacking only	115.71 (97.46)	145.76 (122.76)	30.38 (−46.85, 107.62)
	Control	132.26 (62.67)	152.93 (91.66)	20.66 (−11.70, 53.03)
School day computer games	ST and Snacking	84.70 (177.13)	54.73 (150.16)	−23.52 (− 131.68, 84.62)
	ST only	93.69 (132.15)	32.52 (53.22)	−58.34 (−117.78, 1.08)
	Snacking only	49.00 (47.97)	59.61 (63.65)	6.84 (−32.71, 46.40)
	Control	69.64 (104.26)	59.28 (73.98)	−10.35 (−75.95, 55.24)
Weekend day computer games	ST and Snacking	137.05 (246.39)	88.00 (177.39)	−33.52 (− 148.29, 81.23)
	ST only	107.82 (98.86)	60.04 (88.77)	−42.56 (−88.00, 2.87)
	Snacking only	89.00 (76.79)	88.84 (105.97)	11.46 (−72.98, 95.90)
	Control	110.00 (125.88)	75.00 (102.04)	−35.00 (−92.14, 22.14)
School day smartphone use	ST and Snacking	52.69 (60.78)	58.50 (80.52)	15.38 (−32.36, 63.13)
	ST only	57.66 (95.71)	83.75 (135.38)	15.66 (−6.70, 38.03)
	Snacking only	31.50 (85.10)	11.30 (20.83)	−22.61 (− 67.78, 22.55)
	Control	50.35 (65.94)	46.15 (49.55)	−8.07 (−49.38, 33.23)
Weekend day smartphone use	ST and Snacking	47.50 (56.06)	57.89 (96.02)	31.25 (−24.40, 86.90)
	ST only	49.33 (71.59)	81.00 (112.92)	27.60 (−17.83, 73.03)
	Snacking only	98.35 (36.88)	28.15 (58.51)	−77.76 (−277.11, 121.57)
	Control	51.07 (84.31)	43.46 (49.47)	−11.53 (−65.62, 42.54)

*ST* screen-time

Difference in means were calculated as post-intervention mean minus baseline mean

Little to no changes were found between baseline and post-intervention reports of the dietary variables assessed in any of the groups (see Table 4). Additional file 2: Table S3 shows the baseline, post-intervention and change values for the secondary outcome variables. Changes differed across groups and levels of measures and were small.

**Table 4:** Children’s eating behaviours, and change in eating behaviours by study arm

Eating behaviour variables	Study arm	Mean (SD) frequency/day at baseline (week 0)	Mean (SD) frequency/day at post-intervention (week 13)	Difference in means (95% CI)
Fruit	ST and Snacking	1.76 (0.98)	1.54 (0.91)	− 0.21 (− 0.69, 0.26)
	ST only	3.30 (1.37)	2.94 (1.42)	− 0.36 (− 0.79, 0.08)
	Snacking only	1.82 (1.25)	2.14 (1.51)	0.32 (−0.49, 1.13)
	Control	2.73 (1.38)	2.83 (1.47)	0.10 (−0.29, 0.49)
Vegetables	ST and Snacking	2.80 (1.28)	2.80 (1.41)	0.00 (−0.38, 0.38)
	ST only	3.18 (1.31)	3.20 (1.31)	0.02 (−0.38, 0.42)
	Snacking only	2.67 (1.57)	2.60 (1.48)	−0.07 (− 0.88, 0.74)
	Control	2.50 (1.67)	2.21 (1.38)	−0.17 (−1.09, 0.73)
Savoury snacks	ST and Snacking	1.16 (0.48)	1.04 (0.54)	−0.11 (− 0.32, 0.08)
	ST only	1.06 (0.81)	1.38 (0.91)	0.32 (−0.12, 0.76)
	Snacking only	1.28 (0.95)	1.67 (1.34)	0.39 (−0.13, 0.91)
	Control	1.03 (0.52)	0.86 (0.54)	−0.17 (− 0.36, 0.03)
Sweet snacks	ST and Snacking	1.26 (0.82)	1.42 (1.21)	0.17 (−0.33, 0.66)
	ST only	1.78 (1.04)	1.78 (1.45)	0.00 (−0.52, 0.52)
	Snacking only	1.46 (0.88)	1.32 (0.91)	−0.07 (− 0.24, 0.09)
	Control	1.64 (1.38)	1.67 (1.33)	0.04 (−0.20, 0.27)

Table 5 shows the mean time parents spent in each of the screen-based activities at baseline and post-intervention. Reductions were seen in TV/DVD viewing, computer use, and weekend day smartphone use among parents in some groups, whereas parents’ weekday smartphone use increased in some groups.

**Table 5:** Parent screen-time and change in parent screen-time according to study arm

Screen-time variables	Study arm	Mean (SD) minutes/day at baseline (week 0)	Mean (SD) minutes/day at post-intervention (week 13)	Difference in means (95% CI)
Week day TV/DVD viewing	ST and Snacking *n* = 20	165.00 (155.58)	142.50 (155.99)	−22.50 (−49.78, 4.78)
	ST only *n* = 22	115.90 (71.74)	152.04 (131.93)	34.29 (−19.22, 87.79)
	Snacking only *n* = 12	152.50 (115.45)	125.45 (65.48)	−35.45 (−111.83, 40.93)
	Control *n* = 12	70.00 (43.06)	57.50 (43.30)	−12.50 (−37.49, 12.49)
Weekend TV/DVD viewing	ST and Snacking	210.50 (168.60)	160.50 (108.06)	−50.00 (− 118.14, 18.14)
	ST only	173.55 (106.80)	181.90 (101.47)	5.90 (−27.73, 39.54)
	Snacking only	145.00 (90.30)	174.54 (94.69)	21.81 (−58.34, 101.98)
	Control	92.50 (50.29)	87.50 (56.42)	−5.00 (−39.32, 29.32)
Week day computer games	ST and Snacking	48.75 (109.99)	25.00 (34.41)	−23.75 (−77.03, 29.53)
	ST only	61.19 (46.95)	46.36 (49.62)	−15.48 (−26.39, −4.56)
	Snacking only	42.50 (85.29)	42.50 (80.35)	−0.20 (−-93.51, 88.05)
	Control	22.50 (52.93)	17.50 (37.20)	−5.00 (−42.97, 32.977)
Weekend day computer games	ST and Snacking	62.25 (111.21)	35.50 (56.79)	−26.75 (−79.69, 26.19)
	ST only	103.00 (83.38)	70.91 (71.44)	−32.19 (−57.14, −7.24)
	Snacking only	35.00 (54.02)	67.50 (169.76)	35.45 (−96.34, 167.25)
	Control	40.00 (73.85)	30.00 (54.27)	−10.00 (−52.49, 32.49)
Week day smartphone use	ST and Snacking	67.00 (66.26)	111.00 (265.33)	44.00 (−77.69, 165.69)
	ST only	88.18 (127.63)	153.86 (214.74)	68.81 (−23.61, 161.23)
	Snacking only	72.50 (83.35)	56.25 (52.79)	−23.18 (−57.32, 10.95)
	Control	87.50 (61.96)	60.00 (55.75)	−27.50 (−6.46, 6.46)
Weekend day smartphone use	ST and Snacking	71.50 (79.62)	64.50 (83.88)	−7.00 (−26.68, 12.68)
	ST only	97.72 (129.57)	164.09 (211.80)	70.95 (−20.35, 162.25)
	Snacking only	42.50 (56.43)	61.25 (78.97)	23.18 (−26.05, 72.41)
	Control	103.33 (69.72)	77.50 (65.79)	−25.83 (−66.42, 14.75)

Few changes were found between baseline and post-intervention parental reports of dietary variables assessed in any group (see Table 6).

**Table 6:** Parents’ eating behaviours, and change in eating behaviours by study arm

Eating behaviour variables	Study arm	Mean (SD) frequency/day at baseline (week 0)	Mean (SD) frequency/day at post-intervention (week 13)	Difference in means (95% CI)
Fruit	ST and Snacking	2.27 (1.33)	2.52 (1.52)	0.26 (− 0.31, 0.83)
	ST only	2.17 (1.21)	2.17 (1.21)	0.00 (−0.57, 0.57)
	Snacking only	2.00 (1.31)	2.18 (1.43)	0.00 (−0.84, 0.84)
	Control	2.29 (1.60)	2.46 (1.83)	0.17 (− 0.55, 0.89)
Vegetables	ST and Snacking	2.81 (1.43)	3.05 (1.32)	0.31 (− 0.07, 0.70)
	ST only	2.52 (1.32)	2.81 (1.28)	0.29 (−0.21, 0.79)
	Snacking only	2.77 (1.43)	3.50 (1.05)	0.60 (−0.32, 1.52)
	Control	2.95 (1.51)	2.83 (1.63)	−0.12 (− 0.63, 0.38)
Savoury snacks	ST and Snacking	0.65 (0.54)	0.90 (0.58)	0.24 (0.03, 0.43)
	ST only	0.95 (0.55)	0.90 (0.53)	−0.05 (−0.27, 0.17)
	Snacking only	0.90 (0.58)	0.95 (0.52)	0.05 (−0.31, 0.41)
	Control	0.67 (0.65)	0.75 (0.58)	0.08 (−0.27, 0.43)
Sweet snacks	ST and Snacking	1.32 (1.05)	1.35 (1.03)	0.16 (−0.21, 0.54)
	ST only	1.19 (0.81)	1.21 (1.07)	0.02 (−0.33, 0.38)
	Snacking only	1.13 (0.50)	1.04 (0.52)	−0.20 (− 0.50, 0.10)
	Control	0.79 (0.54)	0.79 (0.54)	0.00 (−0.54, 0.54)

## Discussion

The aim of this paper was to report on the feasibility and potential for efficacy of the Kids FIRST pilot RCT intervention. The results demonstrate initial promise in relation to both the feasibility of recruiting and retaining families to the intervention and the potential for the Kids FIRST intervention to bring about some health behaviour changes. However, given that we could not control for clustering within schools, implications of our findings are still tentative.

In terms of feasibility, this study successfully recruited schools and families into all four study arms and retained them over a period of 13 weeks with a retention rate ≥ 74%. While attrition is commonplace in intervention studies, our loss to follow-up was modest and reasons for participant attrition were primarily logistical (e.g. child absence from school or a lack of time for parents to take part) rather than due to any reported problems experienced with participating in the intervention itself. While these initial recruitment and retention figures are encouraging, we acknowledge that despite targeted efforts to recruit a socioeconomically and ethnically diverse range of families, those who took part were primarily white British, well-educated parents and their children. Further work is required to explore the feasibility of recruiting a more diverse sample of families for a full RCT trial of Kids First. This might require some additional tailoring of the intervention and formative work to understand parental needs in order to meet the specific requirements of these groups. Families (parents and children) demonstrated good compliance with completing study measures at baseline and post-intervention, highlighting initial acceptability of the chosen methods of assessment.

Process evaluation results suggested that only 7% of parents signed up to the study because they were interested in healthy eating compared to 11% who were interested in screen-time and 9% who were interested in physical activity. Such low numbers show the complexity of engaging families in behaviour change when they do not see certain behaviours as concerning. A recent study suggested that receiving messages on the immediate and long-term health risks associated with specific behaviours would help parents reduce these health behaviours in their children [43]. Participatory research methods [44] may be an important design to underpin future interventions targeting multiple health behaviours in young people. Understanding the specific behaviours that young people engage in at home, the outcomes of these behaviours that parents are concerned about, and the behaviours and that parents and children feel are feasible to change, is all key for informing future intervention and health promotion strategies.

Although most parents who received and used resources found them useful, many parents did not use the resources and so additional tailoring of the intervention and formative work to identify additional/different strategies is required to achieve higher levels of engagement with resources relating to the dietary component of the intervention. This might include greater parent and child engagement work early in the development of this aspect of project through a focus on the capability and opportunity elements of the COM-B (Capability, Opportunity, Motivation – Behaviour) framework.

While the current study was not powered to detect statistical changes as a result of the Kids FIRST intervention, it was designed to target screen-time reduction, either alone or alongside changes to snacking. The behaviours typically of concern in the literature, for their negative associations with health outcomes, are TV/DVD viewing and computer gaming [45]. Our preliminary findings indicate that screen-based behaviours were shown to decrease for children in both the screen-time and snacking and the screen-time only intervention groups from pre to post assessment on both school days and weekend days. It would not be desirable to see changes at one time point (e.g., school day) that are reversed and compensated for at another time (e.g., weekend day). Encouragingly, there appears to be no such effect for TV viewing, although the reduction shown by the screen-time group was quite small at weekends. Similar trends were shown for reductions in computer gaming. Overall, therefore, with reductions in TV viewing and computer gaming found over weekdays and weekend days for both screen-time intervention groups, but not for the snacking-only group, the screen-time reduction components of the intervention seem highly feasible and generally successful for modifying the behaviours of TV viewing and computer gaming in 9–11 year old children.

The use of smartphones is an important screen-based behaviour that is still relatively understudied [46], and there is little documented about this from a behaviour change standpoint. However, smartphone ownership and use is increasing in 8–11 year olds [47]. Our initial results show considerable variation as children in both screen-time groups appeared to *increase* their smartphone use during school and weekend days, whereas smartphone use in the snacking-only group decreased. It is likely that self-report methods for assessing smartphone use are inadequate to capture what is a high frequency, short duration, sporadic behaviour. This will make it difficult to capture time estimates of this behaviour. Furthermore, it is possible that the intervention materials weren’t specific enough to target smartphone use (i.e. an evidence-based focus on TV viewing, computer games and tablet use). Improvements to smartphone software which enable users to track their smartphone use are becoming more commonplace and these changes will doubtless enhance the future measurement and reporting of such behaviours.

Overall, similar trends in screen-time behaviours were evident for parents. This was expected and confirms the value in targeting parent and child behaviours. Parent and child screen use behaviour are often highly correlated, and parents act as role models for screen use [30]. Reductions in parent’s weekday and weekend day TV/DVD viewing were found in the screen-time and snacking group, weekday computer game use decreased in all groups, and weekend day computer use for work decreased in the screen-time and snacking, the screen-time only group, and the control group. Smartphone use generally showed a slight increase, as was seen with the children’s data. However, for both the children and parents, some changes in screen use were also seen in the control group which suggests high variability in the behaviour change trends across days and screen-based behaviours. This may also reflect the difficulty in capturing some of these behaviours. In general, the most consistent trends appeared to be for TV viewing and computer gaming where decreases were seen in the screen-time intervention groups, but largely not in the control group.

It is noteworthy that there were little to no changes in any of the dietary variables assessed across the intervention groups. This suggests that the dietary behaviours of children within the family context are more difficult to change. This could be due to many factors, including preferences from other family members, economic influences on purchases, or habits. For the latter, we found few meaningful changes in habit measures. In the screen-time only group, we saw increased reported habit for eating fruit and vegetables while watching TV but eating energy-dense snacks while watching TV also increased in this group. It appears that reductions in habit for dietary intake generally did not take place and therefore the lack of change in eating behaviours is plausibly linked to strong habits. This suggests that future research needs to place greater emphasis on both snacking per se and snacking in front of the TV, and on the factors likely to be successful in changing what appear to be entrenched habits [48]. Similarly, we found little evidence of change in dietary behaviours for parents, and this may have contributed to the lack of change in the eating habits of children. By devising a family-based intervention, Kids FIRST deliberately targeted both child and parent behaviours. While we found improvements in both child and parent screen use, the evidence gained from this pilot work suggests much less impact on eating behaviours.

Social Cognitive Theory suggests that self-efficacy is an important determinant of behaviour change [49]. We found that children’s self-efficacy to reduce their TV/DVD viewing and use of computers increased for those in the ST group, but no other changes in self-efficacy were seen. Of the four social cognitive mechanisms thought to be important sources of self-efficacy, it is plausible that the most likely to affect children’s TV viewing are modelling and encouragement [50]. Therefore, changes in self-efficacy for reducing TV viewing and computer use are likely to require clear parental input through both modelling of appropriate screen-time and personal encouragement for alternative behaviours. Changes in children’s self-efficacy for dietary behaviour were not observed. Again, strong parental input may be required for such changes to occur and further targeting of parental modelling and encouragement could be important for bringing about such dietary changes. Feelings of parental capability as well as general opportunity to create helpful social and physical environments, may be important elements of the COM-B model to consider in future. Moreover, no changes were seen for parental rules regarding screen use or diet, although rules did increase for the screen-time and snacking group. These findings confirm the view that parental guidance and influence were not changed by the intervention or, whatever changes were made were not potent enough. More work is required on how to ensure strong parental engagement in child screen-use and associated dietary practices.

The results of the present feasibility study are encouraging. Strengths of this project include the fact that the Kids FIRST intervention is designed based on review-level evidence and extensive formative work using a range of appropriate theoretical frameworks, and that it involves both parents and children. The use of a family- and school-based approach is a further strength, given evidence that children receive extensive health behaviour messages via schools, and retention in the intervention was very high across conditions. However, there are several shortcomings that need to be considered. The sample consisted of predominantly well-educated white British participants. It is not possible, therefore, to state whether the results would have been similar in a more ethnically and socio-economically diverse sample. Moreover, our sample generally had higher fruit and vegetable intake than reported in previous studies, which might impact generalisability and contribute to the lack of dietary change. As this was a feasibility study that was not powered to detect differences between groups, the analyses we have conducted to compare baseline and follow-up within conditions do not allow consideration of differences between groups for the outcome measures, and thus does not provide reliable evidence of the effectiveness of the intervention. However, the design and analyses of this study are consistent with other feasibility studies and, as highlighted by Jago et al. [51], “it is important that these preliminary studies are conducted and the findings disseminated prior to conducting larger trials”. Screen-time was measured via self-report, given the absence of any more ‘objective’, or device-based, measures, and the ASAQ does not capture multi-screen use (e.g., watching TV whilst using a smartphone). Further work could address this by exploring concurrent screen-use behaviours, which are increasingly common. Seasonal variation could have affected the self-reported data on screen time in particular given that baseline data were collected in late autumn/early winter and follow up was conducted in late winter/early spring. Such variation should be accounted for in larger-scale trials and should be taken into account when interpreting these results. Lastly, the randomisation of schools, and thus children into groups, led to an uneven profile regarding our primary outcomes of ST, and physical environmental factors. For example, for those in the screen-only intervention group only 13% had a TV in their bedroom, whereas for those in the screen/snack group it was 50%. This was exacerbated by challenges with recruiting schools to this programme which meant that we ended up with only one school per arm. Such shortcomings would need targeting in the delivery of any such programmes in future.

While some trends were evident for screen-time changes, dietary behaviours remained largely unchanged, as did habit strength and self-efficacy and parental modelling; all may have contributed to the unpromising results for dietary change. These factors may require a more potent intervention strategy, including real-time self-monitoring and prompting. Furthermore, given the rapid pace in the development of screens, and screen-use, future research addressing issues of measuring screen-use is warranted (e.g., use of wearable cameras).

## Conclusion

In conclusion, this novel pilot intervention has shown promise and value in targeting children and parents, through the home and school, to improve unhealthy snacking and screen use. The Kids First intervention has been shown to be feasible and acceptable to children, parents and teachers. Changes that could be made before a full trial include exploring the feasibility of recruiting a more diverse sample of families and to use this to alter the recruitment strategies for a full RCT trial. In addition, further development work is required to achieve higher levels of engagement with the Kids FIRST resources, and to test further strategies to influence dietary change. This might include greater parent and child engagement work early in the development of the project.

## Supplementary information

**Additional file 1.** Consort Flow diagram.

**Additional file 2: Table S1.** Kids FIRST intervention strategies, behaviour change techniques and links with theoretical constructs. **Table S2.** Kids FIRST intervention blocks and examples of content. **Table S3.** Description and distribution (%) of demographic, individual, behavioural, and home social and physical environmental variables at baseline according to study arm.

## Data Availability

The dataset and protocol supporting the conclusions of this article are available on request of the corresponding author. All materials (questionnaires) used for the purpose of this study are available on request of the corresponding author.

## Abbreviations

ED snacks: Energy-dense snacks
FV: Fruit and vegetables
RCT: Randomised controlled trial
SB: Sedentary behaviour
Sn: Snacking only group
ST: Screen-time
TV: Television viewing
