# Supplementary material for: Reducing screen-time and unhealthy snacking in 9–11 year old children: the Kids FIRST pilot randomised controlled trial
Source: BMC Public Health. 2020 Jan 29;20:122. doi: 10.1186/s12889-020-8232-9 (PMC6988217; doi:10.1186/s12889-020-8232-9)
Supplement: Supplementary file 1 — Additional file 1. Consort Flow diagram. [file 12889_2020_8232_MOESM1_ESM.docx]

Families assessed for eligibility (n=75)

Schools randomized (n=4)

Assessed for primary and secondary outcomes (n=17 children, 13 parents)

Lost to follow-up (n=4 children, 6 parents from ST+Sn; (absent from school / no longer wanting to participate)

Allocated to St+Sn intervention (n=21 children, 19 parents)

♦ Received allocated intervention (n=21 children, 19 parents)

Lost to follow-up (n=4 children, 6 parents (absent from school / no longer wanting to participate)

Allocated to control (n=15 children, 12 parents)

Assessed for primary and secondary outcomes (n=11 children, 6 parents)

## Allocation

## Assessment

## Follow-Up

## Enrollment

Families screened prior to eligibility assessment (n=99)

Families excluded (n=24) due to insufficient numbers providing consent (n < 8 families per school) in three schools

## Screened

Assessed for primary and secondary outcomes (n=11 children, 10 parents)

Assessed for primary and secondary outcomes (n=24 children, 19 parents)

Lost to follow-up (n=1 child, 3 parents from ST only; (absent from school / no longer wanting to participate)

Lost to follow-up n=3 children, n=2 parents from Sn only (absent from school / no longer wanting to participate)

Discontinued intervention (give reasons) (n= )

Allocated to ST only intervention (n=25 children, 22 parents)

♦ Received allocated intervention (n=25 children, 22 parents)

Allocated to Sn only intervention (n=14 children, 12 parents)

♦ Received allocated intervention (n=14 children, 12 parents)
