# Supplementary material for: Reducing screen-time and unhealthy snacking in 9–11 year old children: the Kids FIRST pilot randomised controlled trial
Source: BMC Public Health. 2020 Jan 29;20:122. doi: 10.1186/s12889-020-8232-9 (PMC6988217; doi:10.1186/s12889-020-8232-9)
Supplement: Supplementary file 2 — Additional file 2: Table S1. Kids FIRST intervention strategies, behaviour change techniques and links with theoretical constructs. Table S2. Kids FIRST intervention blocks and examples of content. Table S3. Description and distribution (%) of demographic, individual, behavioural, and home social and physical environmental variables at baseline according to study arm. [file 12889_2020_8232_MOESM2_ESM.docx]

**Supplementary Table 1.** Kids FIRST intervention strategies, behaviour change techniques and links with theoretical constructs.

| Social ecological model level | Potential mediators | Behaviour Change Technique | Examples of Kids FIRST intervention strategy | Theory | Kids FIRST intervention component(s) |
| --- | --- | --- | --- | --- | --- |
| Individual | Habit | - Habit formation - Role play - Goal setting - Self-monitoring | - Setting goals for alternative behaviours that are domain and context specific - Encourage parents and children to identify current habits and cues - Encourage and promote frequent and consistent repetition of the new behaviour in the same situational context - Encourage and promote routinisation of alternative behaviours | HT | Introductory session  Online session  Habit booklet  Goal setting sheets  In-class lesson  Prompts (email / text / post)  Website blog  Monitoring chart  A-Z directory of alternative behaviours |
|  | Self-efficacy | - Prompt barrier identification - Goal setting - Self-monitoring - Social processes of encouragement and support | - Identify and challenge barriers to reducing unhealthy eating at screens - Provide example or case studies of peers (similar aged role models) not eating unhealthy snacks in front of screens - Enactive self-mastery – activities where we encourage parents and children to break the behaviour down into smaller steps that are easier to overcome; provide examples of alternative situations and behaviours that offer rewards (family meal times) | SCT  BCT | Introductory session  Online session  Newsletter  Goal setting sheets  In-class lesson  Prompts (email / text / post)  Website blog  Monitoring chart  A-Z directory of alternative behaviours |
| Behavioural | Parent Screen-time (modelling) | - Prompt identification as a role model - Provide opportunities for social comparison - Goal setting | - Provide case studies where parents have successfully reduced screen-time or unhealthy snacking (group dependent) - Encourage healthy role modelling - Provide examples of alternative behaviours and prompt parents to participate in alternative behaviours with their child - Time budgeting - Provide opportunity and encouragement for parents to set goals that are specific to them and their families | SCT  BCT | Online sessions  Newsletters  A-Z directory  Goal setting sheets  Website blog |
|  | Child behaviour (screen-time / unhealthy snacking – group dependent) | - Prompt identification as a role model - Provide opportunities for social comparison - Goal setting | - Provide case studies of excessive screen-time/unhealthy snacking and some consequences that are relevant to the age group - Encourage children to role model (to parents and friends) with interactive examples (games and class lessons) - Provide examples of alternative behaviours and encourage child to participate in active alternative behaviours together with family / friends - Time budgeting - Provide opportunity and encouragement for children to set goals that are specific to them | SCT  BCT | In class lessons  Homework activities  A-Z directory  Goal setting sheets  Newsletters |
| Social | Parent rules / limits | - Provide opportunities for social comparison - Goal setting | - Provide opportunity and encouragement for parents to think of and set appropriate rules / limits for their household - Provide case-studies and examples of rules and limits - Promote discussion about rules and limits | SCT | Online sessions  Newsletters  Website blog and resources  Goal setting sheets |
| Environment | Accessibility and availability of screens / snacks | - Environmental changes - Prompt barrier identification | - Informing parents of the link between availability and accessibility and screen-time and unhealthy snacking (group dependent) - Provide opportunity and encouragement for parents and children to come up with ideas on how to limit access and availability of screens and unhealthy snack foods(group dependent) - Provide examples and case studies of ‘success’ - Provide alternative behaviours and instructional tips on how to make alternative activities and games more available and accessible at home | SCT  BCT | Face-to-face sessions  Newsletters  In-class lessons  A-Z of alternative behaviours  Website blog and resources |
|  | Screens in bedroom | - Environmental changes - Prompt barrier identification | - Informing parents of the link between screens in bedrooms and screen-time and unhealthy snacking - Providing opportunity for parents to discuss ways of removing screens from bedrooms (or limiting their use) - Provide parents with case studies of ‘success’ - Encourage parents to set goals | SCT  BCT | Face-to-face sessions  Newsletters  In-class lessons  A-Z of alternative behaviours  Website blog and resources |

Theory: HT (Habit Theory); SCT (Social Cognitive Theory); BCT (Behavioural Choice Theory);

**Supplementary Table 2.** Kids FIRST intervention blocks and examples of content

| **Block** | **Potential mediator targeted** | **Week** | **In school** | **At home** |
| --- | --- | --- | --- | --- |
|  | | **0** | **Introductory session:**   - Overview of project and rationale - Schedule of sessions and when to expect resources - Website details and login information - Parents shown goals (specific to each group, e.g. Group 2 goal: ‘reduce my own and my child’s screen-time’) - Parents given resources including an a-z directory of alternative behaviours and/or snacks (all groups), an activity jar with alternative activity/snack ideas (all groups), and recipe cards (group 1 and 3 only) | |
| **1** | Habit and self-efficacy | **1** |  | **Online session 1: Habits and self-efficacy**   - PowerPoint slideshow on Habit formation - Habit formation booklet (received and talked through at introductory session and based on previous research [[52](#_ENREF_52)]) - Parents to consider current habits and cues to overcome/form new habits - Examples of success stories - Breaking behaviours down into small steps - Monitoring encouraged |
|  |  | **2** | **Lesson 1: habits and confidence**   - What are habits? - What habits do we have that are good or not so good? - Knowledge of screen-time and snacking recommendations - Alternative behaviours |  |
|  |  | **3** | **Newsletters and resources sent home** | |
| **2** | Role modelling | **4** |  | **Online session 2: Role modelling**   - PowerPoint slideshow on role modelling - Top tips for being a successful role model - Encouraging and educating on alternative behaviours |
|  |  | **5** | **Lesson 2: Invent a recipe / game**   - Children in group 1 and 3 were encouraged to create a healthy snack from a list of ingredients - Children in group 2 were encouraged to come up with a new game to play instead of using screens after school - Discussions about alternative snacks and behaviours |  |
|  |  | **6** | **Newsletters and resources sent home** | |
| **3** | Home accessibility and availability | **7** |  | **Online session 3: Home availability and accessibility**   - PowerPoint slideshow on home availability and accessibility - Audio recording of expert advice on availability and accessibility - Examples of success stories - Alternate behaviours / snacks encouraged - Smart shopping promoted (Group 1 and 3 only) |
|  |  | **8** | **Lesson 3: Guess who**   - Children were in pairs with a piece of paper with 9 snacks / activities. In their pairs they had to describe and ask questions to guess the snack/activity that their partner had chosen. - Discussions on healthy/unhealthy - Discussions on availability and accessibility at home |  |
|  |  | **9** | **Newsletters and resources sent home** | |
| **4** | Rule for screen-time/snacking | **10** |  | **Online session 4: Parental / home rules**   - Audio recording of expert advice on rules at home regarding screen-time (group 1 and 2), and healthy snacking (group 1 and 3) - Top tips sheets - Activity / snack jar encouraged |
|  |  | **11** | **Lesson 4: Time use / sorting activities**   - Groups 1 and 2 children were asked to use pictures of clocks to depict the time spent before and after school using screens. Total time was calculated and compared in classes. - Groups 1 and 2 children: activity covering ‘what else could you use this time for’, and ‘who/what (parents/rules) would need to help you swap this time’ - Group 3 children played a sorting game in groups. Sorting fruit and vegetables into a variety of groups. - Group 3 children then discussed when, where and how they could swap some of the unhealthy snacks they ate with fruit and vegetables |  |
|  |  | **12** | **Newsletters and resources sent home** | |

**Supplementary Table 3.** Description and distribution (%) of demographic, individual, behavioural, and home social and physical environmental variables at baseline according to study arm

|  | **Study arm** | **Mean (SD) baseline (week 0)** | **Mean (SD) post-intervention (week 13)** | **Difference in means (95% CI)** |
| --- | --- | --- | --- | --- |
| **Secondary outcomes** |  |  |  |  |
| **Individual** |  |  |  |  |
| ***Habits*** |  |  |  |  |
| Habit for watching television |  |  |  |  |
|  | ST and Snacking | 8.47 (2.54) | 9.19 (2.78) | 0.73 (-0.43, 1.90) |
|  | ST only | 8.44 (2.90) | 7.56 (2.13) | -0.87 (-2.04, 0.31) |
|  | Snacking only | 8.71 (2.64) | 8.92 (2.58) | 0.08 (-1.12, 1.27) |
|  | Control | 7.66 (8.14) | 8.14 (2.14) | 0.38 (-0.14, 0.91) |
| Habit for eating snack foods while watching TV |  |  |  |  |
|  | ST and Snacking | 9.61 (2.88) | 10.61 (1.99) | 1.10 (-0.33, 2.54) |
|  | ST only | 10.16 (2.35) | 10.54 (1.93) | 0.47 (-0.51. 1.47) |
|  | Snacking only | 9.64 (3.20) | 9.77 (3.03) | 0.31 (-0.75, 1.37) |
|  | Control | 9.00 (2.33) | 9.29 (2.72) | 0.23 (-1.21, 1.67) |
| Habit for eating fruit and vegetables while watching TV |  |  |  |  |
|  | ST and Snacking | 10.33 (2.19) | 10.75 (2.02) | 0.32 (-0.56, 1.19) |
|  | ST only | 8.76 (2.27) | 9.87 (1.98) | 1.13 (0.14, 2.11) |
|  | Snacking only | 9.79 (2.48) | 10.14 (2.98) | 0.08 (-1.07, 1.22) |
|  | Control | 9.57 (2.37) | 10.08 (2.25) | 0.69 (-0.66, 2.15) |
| Habit for eating snack foods |  |  |  |  |
|  | ST and Snacking | 10.95 (1.46) | 11.05 (1.64) | 0.21 (-0.54, 0.96) |
|  | ST only | 10.48 (1.82) | 9.33 (2.09) | -1.04 (-2.14, 0.32) |
|  | Snacking only | 9.79 (2.32) | 9.57 (2.71) | -0.31 (-1.28, 0.66) |
|  | Control | 9.67 (2.47) | 10.07 (2.19) | 0.15 (-0.26, 0.57) |
| Habit for eating fruit and vegetables |  |  |  |  |
|  | ST and Snacking | 9.38 (2.13) | 10.09 (2.17) | 0.68 (-0.18, 1.55) |
|  | ST only | 8.00 (2.48) | 8.42 (2.68) | 0.78 (-0.51, 2.07) |
|  | Snacking only | 9.79 (2.26) | 10.00 (2.45) | 0.08 (-0.82, 0.98) |
|  | Control | 9.07 (2.12) | 8.57 (2.14) | -0.62 (-1.55, 0.32) |
| ***Self-efficacy*** |  |  |  |  |
| Self-efficacy for reducing time spent watching TV/DVD’s or using computers |  |  |  |  |
|  | ST and Snacking | 13.71 (2.78) | 14.19 (2.71) | 0.48 (-0.72, 1.68) |
|  | ST only | 12.96 (2.97) | 14.16 (2.49) | **1.41 (0.22, 2.60)** |
|  | Snacking only | 13.78 (3.21) | 13.61 (2.32) | -0.17 (-1.79, 1.46) |
|  | Control | 11.79 (3.17) | 12.62 (2.36) | 0.69 (-0.91, 2.29) |
| Self-efficacy for not eating snack foods when watching TV/DVD’s |  |  |  |  |
|  | ST and Snacking | 15.05 (3.01) | 14.67 (3.32) | -0.38 (-1.29, 0.53) |
|  | ST only | 15.32 (2.76) | 15.50 (2.77) | -0.05 (-1.22, 1.12) |
|  | Snacking only | 14.86 (4.05) | 14.42 (3.85) | -0.42 (-4.03, 3.19) |
|  | Control | 13.92 (3.15) | 14.36 (3.02) | 0.54 (-0.58, 1.66) |
| Self-efficacy for increasing fruit and vegetable consumption |  |  |  |  |
|  | ST and Snacking | 14.48 (2.92) | 13.52 (3.93) | -0.95 (-2.87, 0.97) |
|  | ST only | 14.40 (3.35) | 14.84 (2.73) | 0.68 (-1.06, 2.42) |
|  | Snacking only | 14.43 (2.34) | 14.39 (3.09) | 0.01 (-1.60, 1.60) |
|  | Control | 14.00 (2.51) | 14.57 (2.84) | 0.62 (-1.60, 2.82) |
| Self-efficacy for reducing energy-dense snack food consumption |  |  |  |  |
|  | ST and Snacking | 13.90 (3.56) | 14.38 (3.39) | 0.47 (-0.55, 1.51) |
|  | ST only | 13.61 (3.34) | 14.40 (3.30) | 0.91 (-0.31, 2.12) |
|  | Snacking only | 14.61 (2.98) | 14.84 (3.41) | 0.42 (-1.51, 2.34) |
|  | Control | 12.85 (3.91) | 13.71 (2.67) | 0.85 (-0.75, 2.44) |
| **Behavioural** |  |  |  |  |
| Eating dinner while watching TV |  |  |  |  |
|  | ST and Snacking | 1.67 (2.36) | 1.85 (2.21) | 0.11 (-0.69, 0.91) |
|  | ST only | 1.78 (2.54) | 2.00 (2.52) | 0.43 (-0.83, 1.69) |
|  | Snacking only | 2.25 (2.84) | 2.42 (2.89) | 0.33 (-0.88, 1.54) |
|  | Control | 2.33 (2.69) | 2.53 (3.17) | 0.55 (-0.35, 1.44) |
| Eating fruit and vegetables while watching TV |  |  |  |  |
|  | ST and Snacking | 0.76 (0.58) | 0.53 (0.62) | -0.25 (-0.51, 0.01) |
|  | ST only | 0.52 (0.57) | 0.54 (0.56) | 0.05 (-0.21, 0.31) |
|  | Snacking only | 0.50 (0.92) | 0.50 (0.91) | 0.00 (-0.72, 0.72) |
|  | Control | 1.04 (0.77) | 0.92 (0.57) | 0.14 (-0.20, 0.48) |
| Eating energy-dense snacks while watching TV |  |  |  |  |
|  | ST and Snacking | 0.61 (0.47) | 0.55 (0.76) | -0.06 (-0.39, 0.28) |
|  | ST only | 0.49 (0.54) | 0.69 (0.66) | **0.29 (0.03, 0.54)** |
|  | Snacking only | 0.62 (0.69) | 0.67 (0.69) | 0.02 (-0.03, 0.07) |
|  | Control | 0.98 (0.82) | 1.00 (0.84) | 0.11 (-0.38, 0.61) |
| **Social environment** |  |  |  |  |
| Parental rules for TV use |  |  |  |  |
|  | ST and Snacking | 1.76 (0.35) | 1.89 (0.27) | 0.13 (-0.07, 0.34) |
|  | ST only | 1.88 (0.21) | 1.81 (0.32) | 0.00 (-0.17, 0.17) |
|  | Snacking only | 1.81 (0.30) | 1.75 (0.32) | -0.04 (-0.14, 0.06) |
|  | Control | 1.83 (0.30) | 1.79 (0.25) | -0.07 (-0.25, 0.12) |
| Parental rules for computer use |  |  |  |  |
|  | ST and Snacking | 1.86 (0.20) | 1.92 (0.18) | **0.11 (0.01, 0.22)** |
|  | ST only | 1.85 (0.24) | 1.83 (0.32) | -0.02 (-0.13, 0.09) |
|  | Snacking only | 1.79 (0.37) | 1.75 (0.37) | -0.04 (-0.14, 0.06) |
|  | Control | 1.64 (0.38) | 1.74 (0.35) | 0.03 (-0.20, 0.27) |
| Parental rules for energy-dense snack consumption |  |  |  |  |
|  | ST and Snacking | 1.87 (0.23) | 1.92 (0.17) | 0.07 (-0.05, 0.19) |
|  | ST only | 1.95 (0.15) | 1.83 (0.29) | -0.09 (-0.17, 0.01) |
|  | Snacking only | 1.93 (0.19) | 1.93 (0.14) | -0.04 (-0.14, 0.06) |
|  | Control | 1.95 (1.25) | 1.93 (1.44) | -0.07 (-0.17, 0.03) |
| Parental rules for fruit and vegetable consumption |  |  |  |  |
|  | ST and Snacking | 1.20 (0.30) | 1.20 (0.27) | 0.07 (-0.12, 0.25) |
|  | ST only | 1.33 (0.36) | 1.21 (0.29) | -0.12 (-0.30, 0.06) |
|  | Snacking only | 1.35 (0.33) | 1.43 (0.40) | 0.08 (-0.16, 0.33) |
|  | Control | 1.17 (0.36) | 1.17 (0.33) | 0.07 (-0.08, 0.22) |
| Parental rules for eating at the TV |  |  |  |  |
|  | ST and Snacking | 1.84 (0.29) | 1.83 (0.32) | 0.07 (-0.04, 0.17) |
|  | ST only | 1.82 (0.35) | 1.65 (-0.12) | -0.12 (-0.29, 0.04) |
|  | Snacking only | 1.69 (0.38) | 1.74 (0.36) | 0.21 (-0.09, 0.50) |
|  | Control | 1.74 (0.37) | 1.74 (0.39) | -0.17 (-0.52, 0.19) |
| **Physical environment** |  |  |  |  |
| Television in bedroom (% yes) |  |  |  |  |
|  | ST and Snacking | 50 | 53 |  |
|  | ST only | 13 | 21 |  |
|  | Snacking only | 14 | 29 |  |
|  | Control | 50 | 50 |  |
| Computer in bedroom (% yes) |  |  |  |  |
|  | ST and Snacking | 67 | 53 |  |
|  | ST only | 48 | 58 |  |
|  | Snacking only | 36 | 29 |  |
|  | Control | 57 | 50 |  |
| Number of TV screens child has access to at home |  |  |  |  |
|  | ST and Snacking | 1.8 (0.95) | 1.67 (1.02) | -0.15 (-0.17, 0.07) |
|  | ST only | 1.64 (0.81) | 1.83 (0.96) | 0.16 (-0.13, 0.45) |
|  | Snacking only | 1.93 (1.07) | 1.86 (1.03) | -0.07 (-0.10, 0.21) |
|  | Control | 1.79 (1.05) | 1.85 (1.03) | 0.10 (-0.12, 0.33) |
| Number of computer screens child has access to at home |  |  |  |  |
|  | ST and Snacking | 2.25 (1.41) | 1.76 (1.26) | -0.53 (-0.78, 0.12) |
|  | ST only | 2.32 (1.22) | 2.83 (2.69) | 0.51 (-0.16, 0.63) |
|  | Snacking only | 2.21 (1.48) | 1.79 (1.25) | -0.43 (-1.01, 0.46) |
|  | Control | 2.29 (1.64) | 2.14 (1.35) | -0.15 (-1.11, 0.50) |
| Home accessibility of energy-dense snack foods |  |  |  |  |
|  | ST and Snacking | 3.25 (1.25) | 3.00 (1.26) | -0.26 (-0.76, 0.24) |
|  | ST only | 3.72 (0.89) | 3.71 (0.99) | -0.04 (-0.44, 0.36) |
|  | Snacking only | 3.14 (1.09) | 3.35 (0.74) | -0.08 (-0.66, 0.49) |
|  | Control | 2.92 (1.07) | 3.21 (0.89) | 0.28 (-0.24, 0.81) |
| Home accessibility of fruit and vegetables |  |  |  |  |
|  | ST and Snacking | 9.15 (1.81) | 9.00 (1.86) | -0.16 (-0.74, 0.42) |
|  | ST only | 9.08 (1.79) | 9.54 (1.64) | 0.52 (-0.02, 1.07) |
|  | Snacking only | 8.31 (1.70) | 8.77 (1.74) | 0.50 (-0.53, 1.53) |
|  | Control | 9.50 (1.34) | 9.29 (1.38) | -0.21 (-0.93, 0.50) |
